# The Long and Winding Road of Atrial Septostomy

**DOI:** 10.3390/diagnostics10110971

**Published:** 2020-11-19

**Authors:** Julio Sandoval

**Affiliations:** Instituto Nacional de Cardiologia de Mexico, Mexico City 14080, Mexico; julio.sandoval@cardiologia.org.mx

Almost forty years have elapsed since the first description of the use of atrial septostomy in the setting of pulmonary arterial hypertension (PAH) [1]. However, its path towards wide recognition as a useful interventional therapeutic modality for advanced PAH has not been easy. Initially, it was considered a risky procedure. Indeed, the early unacceptable procedure-related mortality has been significantly reduced due to a better selection of candidates, technical improvements, and growing experience in dedicated centers [2]. Despite the use and demonstrated short-term hemodynamic benefits of atrial septostomy in a meta-analysis [3], its long-term safety, efficacy, and therapeutic role in the setting of advanced PAH were not well defined. A relatively frequent outcome of the procedure, the defect’s spontaneous closure, hindered this knowledge [2]. In the last few years, several attempts have been made to avoid this problem using dedicated devices, and stenting the septostomy is one of them [4,5].

The article entitled “Outcomes of Atrioseptostomy with Stenting in Patients with Pulmonary Arterial Hypertension from a Large Single-Institution Cohort,” by Prof. Dr. Sergey V. Gorbachevsky et al., published in the special issue of this journal, adds to this knowledge [6]. In this exciting and well-documented study, the authors performed a retrospective analysis of their considerable experience with atrial septostomy plus stenting. They focused their research mainly on the long-term survival (SV) of sixty-eighty idiopathic PAH patients, thus representing the most extensive series from a single institution reported to date on this dual intervention. Patients were stratified as intermediate and high-risk groups according to the risk stratification of the 2015 guidelines for diagnosis and treatment of PAH from the European Society of Cardiology [7]. Based on their results, the authors conclude that atrial septostomy plus stenting is a safe and effective procedure for the management of PAH patients and that patients in the intermediate-risk group benefit more from the intervention than patients in the high-risk group as they showed better results in terms of functional class, a six min walk test, and long-term SV after the procedure.

Apart from being the most extensive series with the most extended follow-up, this study’s results provide other essential messages. They confirm that atrial septostomy plus stenting is relatively safe (their procedure-related mortality was very low). Most importantly, maintaining the septostomy in the long-term is an attainable goal without adding an extra cost in terms of risk [4]. They confirm the immediate hemodynamic benefits of septostomy and suggest that these results are maintained in the mid-term. Finally, the results of the study give support to the concept that intervening earlier is better and that atrial septostomy can be combined with specific therapy [8].

From this experience and from many other studies, it is clear that atrial septostomy, with or without stenting, does not stop the progression of pulmonary vascular disease in the long-term. However, the procedure temporarily improves patients’ condition. It gives them time to find additional opportunities, such as access to current approved PAH-specific therapy if this was not previously available, or lung transplantation.

Once the problem of long-term patency demonstrated by this and other devices [4,5,6] appears to be solved, atrial septostomy continues its long and winding road to remain an additional strategy for the right ventricular treatment failure from severe PAH. Several reasons still justify its use: (1) the harmful impact of right ventricular failure on patient survival; (2) the unpredictable response to medical treatment. (3) the limited access to lung transplantation; and (4) the disparity in the availability of these treatments throughout the world.

For now, atrial septostomy with or without dedicated devices should only be performed in centers widely experienced in both interventional cardiology and pulmonary hypertension. A more widespread utilization of this useful intervention remains a challenge.
